# Short-Term Changes in Depressive Symptoms Among Patients with Alzheimer’s Disease Following a Precision Medicine Intervention

**DOI:** 10.3390/brainsci16010002

**Published:** 2025-12-19

**Authors:** Rammohan V. Rao, Alan Boyd, Sho Okada, William Lipa, Lance Kelly, Christine Coward, Aida L. Bredesen, Julie Gregory, Dale E. Bredesen

**Affiliations:** 1 Apollo Health, Burlingame, CA 94010, USA; 2 CNS Vital Signs, Morrisville, NC 27560, USA; 3 Pacific Neuroscience Institute, Santa Monica, CA 90404, USA

**Keywords:** Alzheimer’s disease, cognitive decline, therapeutics, PHQ9, depression, ReCODE, diet, lifestyle

## Abstract

**Background/Objectives**: The ReCODE (Reversal of Cognitive Decline) protocol, a precision medicine program for patients with Alzheimer’s disease (AD), has demonstrated promising results in improving cognitive function. However, its impact on mood, specifically depressive symptoms, has been less explored. Depression is highly prevalent in individuals with mild cognitive impairment and AD, shares common risk factors (e.g., systemic inflammation), and negatively affects quality of life and disease trajectory. This study evaluated whether participation in the ReCODE program is associated with measurable improvement in depressive symptoms, as assessed by the Patient Health Questionnaire-9 (PHQ-9). **Methods**: This retrospective, observational analysis utilized de-identified data from 170 individuals diagnosed with mild to moderate AD enrolled in the ReCODE program. Participants received initial consultations and program orientation. Follow-up visits occurred 31 days post-enrollment, including further guidance on diet, lifestyle, medications, and supplements, along with mood assessment using the PHQ-9. Pre- and post-intervention PHQ-9 scores were analyzed using the non-parametric Wilcoxon signed-rank test. **Results**: Participants showed a statistically and clinically significant reduction in PHQ-9 scores. Improvement was observed across all baseline depression categories (mild, moderate, and severe). Critically, the proportion of participants scoring above the clinical threshold for depression decreased substantially following the intervention. **Conclusions**: These findings suggest that the ReCODE precision-medicine framework offers concurrent benefits for both mood and cognition. Extending prior work, our results indicate that the ReCODE program not only benefits cognitive and biological measures but also significantly alleviates depressive symptoms. While the results highlight ReCODE’s potential as a successful multifaceted therapeutic approach addressing both cognitive decline and mental health in Alzheimer’s disease, given the retrospective, uncontrolled design, the findings should be interpreted as associative and hypothesis-generating rather than causal.

## 1. Introduction

Alzheimer’s disease (AD), a neurodegenerative disorder marked by progressive cognitive decline, remains a leading cause of morbidity in aging populations worldwide [1,2,3]. Traditional monotherapeutic, pharmaceutical approaches have yielded limited clinical benefits [4,5]. Recent advances in precision medicine underscore the need to move beyond single-target strategies toward comprehensive, systems-level interventions that address the multiple drivers of neurodegeneration. Thus, we and others have demonstrated that a range of metabolic abnormalities—including insulin resistance, chronic inflammation, hypovitaminosis D, hormonal imbalances, and elevated homocysteine levels—are key contributors to the pathophysiology of AD [6,7,8,9,10]. These metabolic dysfunctions often interact and compound over time, creating biochemical and physiological disturbances that drive cognitive decline [6,7,8,9,10,11].

Recent clinical trials and observational studies have shown that addressing multiple such factors concurrently leads to superior outcomes compared to targeting a single variable [6,10,12,13,14,15,16,17,18,19]. Accordingly, multifactorial therapeutic models have emerged as promising frameworks to both slow and potentially reverse cognitive decline. A multi-therapeutic, personalized approach that evaluates and treats the full range of contributing metabolic factors may be more aligned with the multifactorial nature of AD [6,10,12,13,14,15,16,17,18,19]. This strategy aims not just to manage symptoms but to alter the trajectory of disease progression by restoring systemic balance and supporting neuronal resilience. Moreover, this approach is more sustainable in the long term as it promotes overall health and addresses comorbidities that commonly accompany aging and cognitive decline, such as hypertension, insulin resistance, and dyslipidemia [7,10,20,21,22,23,24].

The ReCODE (Reversal of Cognitive Decline) program is a multi-therapeutic, precision medicine approach that evaluates and treats the full range of contributing metabolic, infectious, immune, vascular, and toxic factors. The program targets multiple biological mechanisms underlying AD—such as inflammation, insulin resistance, nutrient deficiencies, toxin exposure, and hormonal imbalances—through personalized lifestyle and therapeutic interventions [6,25]. Our prior study provided an in-depth overview of these upstream drivers and demonstrated improvement in both cognitive and systemic biomarkers following implementation of the ReCODE protocol [25]. Notably, the first clinical trial using this protocol demonstrated statistically significant cognitive improvement in 84% of patients with mild cognitive impairment or early-stage dementia over a 9-month intervention, measured by MoCA and other neuropsychological tools [7,20]. Complementing this trial, other independent observational studies and clinical trials have validated the benefits of multi-therapeutic approaches that evaluate and treat a range of contributing factors in varied clinical settings [10,15,16,17,18,19,21,22,23,24]. Across these studies, cognitive benefits were observed through standardized assessments such as the MoCA and CNS Vital Signs, while complementary measures including MRI volumetrics and laboratory analyses demonstrated concurrent improvements in metabolic health and quality of life along with reduced reliance on medications [6,10,12,13,14,15,16,17,18,19,21,22,23,24].

Depression is a frequent comorbidity in AD and related dementias, affecting up to 50% of patients [26,27]. It contributes to decreased quality of life, increased caregiver burden, and potentially accelerated cognitive decline [28,29,30,31,32,33,34,35]. According to the ICD-11, the key symptom domains for depression include persistent low mood, a markedly diminished interest or pleasure in activities, reduced ability to concentrate, and feelings of worthlessness or excessive guilt. Other key symptoms are changes in sleep or appetite, fatigue or loss of energy, psychomotor changes, and recurrent thoughts of death or suicidal ideation [36]. The Patient Health Questionnaire-9 (PHQ-9) is a validated instrument widely used to assess the severity of depressive symptoms and to monitor clinical change [28,29,30]. The PHQ-9 questions cover the key symptom domains of depression, mentioned in the ICD-11. Given the bidirectional relationship between mood and cognition, evaluating depression outcomes is important for understanding the program’s broader therapeutic effects [37]. Depressive symptoms can exacerbate cognitive decline, while cognitive improvement and metabolic restoration may, in turn, improve mood—making mood assessment an important complementary endpoint [38,39].

The present study extends our prior ReCODE findings by focusing specifically on short-term changes in PHQ-9 scores following short-term participation in the ReCODE program. The primary aim was to determine whether the multi-therapeutic program can meaningfully reduce depression in individuals with AD. Accordingly, this study was designed as a focused retrospective analysis of short-term changes in depressive symptoms, complementing our prior ReCODE publications that have reported cognitive, biomarker, and neuroimaging outcomes.

## 2. Materials and Methods

### 2.1. ReCODE Protocol Components, Software and Program Implementation

The ReCODE program (Reversal of Cognitive Decline) is a multifaceted, personalized intervention aimed at mitigating cognitive decline and enhancing brain function. It employs a precision medicine approach, tailoring interventions based on individual biochemical, genetic, and lifestyle risk factors associated with neurodegeneration. While primarily designed for individuals with subjective cognitive impairment (SCI), mild cognitive impairment (MCI), and early-stage dementia associated AD, some individuals at more advanced stages have also reported clinical improvements underscoring ReCODE’s applicability across varying severities of cognitive decline [6,13,25].

The program utilizes an extensive and integrative data set, considerably broader than that used in conventional dementia assessments, to uncover the underlying factors contributing to cognitive decline. These upstream influences may include infections, gut microbiome imbalances, sleep disturbances, toxic exposures, hormonal insufficiencies, and other physiological or metabolic abnormalities. Comprehensive information from laboratory analyses, detailed medical questionnaires, and cognitive assessments is compiled and processed through a specialized algorithm designed to evaluate potential drivers such as inflammation, insulin resistance, nutrient or hormonal deficiencies, pathogenic burden, and genetic susceptibility thereby allowing categorization of participants according to their predominant contributing subtypes of AD [13,40]. Participants receive detailed guidance from their trained physicians and healthcare teams to help them to address these contributing factors systematically. Based on this analysis, participants receive individualized reports outlining the factors most relevant to their condition, along with guidance for further evaluation and management in consultation with their healthcare providers [6,13,25].

In addition to identifying these contributors, the ReCODE approach emphasizes seven core diet and lifestyle principles aimed at supporting neural repair, brain and systemic health. These include adherence to a plant-rich, mildly ketogenic diet emphasizing low-mercury, wild-caught seafood and pastured eggs; incorporation of daily fasting periods; a balanced exercise regimen combining aerobic and resistance training while minimizing sedentary time; consistent restorative sleep through good sleep hygiene and treatment of sleep-related issues such as nocturnal hypoxemia; structured stress-reduction practices involving breathwork and meditation; regular cognitive stimulation and social engagement; toxin avoidance with strategies to enhance detoxification; and tailored nutritional supplementation based on laboratory findings [6,13,25]. This multifactorial structure allows simultaneous targeting of multiple upstream mechanisms driving neurodegeneration, offering a reproducible, data-driven framework that has demonstrated cognitive and metabolic benefits across several independent studies [7,41]. Interestingly, most of the ReCODE components are directly relevant to mood regulation and are therefore particularly pertinent to the outcomes examined in this study.

Notably, no single causative factor was observed in isolation; rather, several interrelated contributors were identified in all participants [6,7,25]. To support implementation and adherence, participants were encouraged to engage regularly with a health coach, with the dual aim of halting further decline and promoting cognitive recovery while enhancing general well-being [6,7,25].

### 2.2. Patient Health Questionnaire-9 (PHQ-9)

The primary outcome assessed in this study was the severity of depressive symptoms, evaluated using the Patient Health Questionnaire-9 (PHQ-9), which is a self-administered, 9-item standard assessment point in depression trials [28,29,30] that is frequently used to monitor depression severity over time and treatment outcomes [28]. Each item is rated on a 4-point Likert scale from 0 (“not at all”) to 3 (“nearly every day”); for example, participants are asked, “Over the last two weeks, how often have you felt little interest or pleasure in doing things?” Scores for all nine items are summed to yield a total score ranging from 0 to 27, with higher scores reflecting greater depressive symptom severity [42]. In this study, PHQ-9 scores were analyzed as continuous variables, with standard thresholds used descriptively to categorize depression severity. Extensive validation studies have confirmed the PHQ-9’s robust psychometric properties, high internal consistency and test–retest reliability [43,44,45]. Furthermore, it has been shown to be a responsive and reliable measure for detecting changes in symptom severity in clinical trials [46,47].

### 2.3. Study Design, Participants, Assessment and Statistical Analysis

This study is a retrospective, observational analysis of de-identified data obtained from individuals with a clinical diagnosis of mild-to-moderate Alzheimer’s disease that were enrolled in the ReCODE program [6,25]. Patients who were diagnosed with subjective cognitive impairment (SCI), mild cognitive impairment (MCI), or early-stage Alzheimer’s disease (AD) by their practitioners and who voluntarily enrolled in the ReCODE program were included in this analysis. Individuals with major medical illnesses such as cardiovascular disease or cancer and pregnant women were excluded. Each participant worked closely with a ReCODE-trained physician, health coach, nutritionist, and other allied practitioners as needed. The multidisciplinary protocol facilitated continuous communication, coordinated care, and personalized support among all members of the clinical team. In general, participants were free of significant comorbidities, and about 70% carried at least one ApoE4 allele, a major genetic risk factor for AD [48,49]. The cohort included an equal number of males and females, with a mean age of 75 years.

Participants underwent initial consultations with clinical practitioners and received a structured orientation to the program protocol [6,25]. Participants also received education and guidance on stress-reduction techniques, including mindfulness and breathing exercises, as part of the ReCODE implementation. Follow-up visits were conducted 31 days post-enrollment, during which participants received further guidance on dietary interventions, lifestyle modifications, pharmacological treatments, and nutritional supplements.

At these visits, mood was also evaluated using the Patient Health Questionnaire-9 (PHQ-9). For the present study, to cover mild symptoms, improve result sensitivity, and ensure study feasibility, a PHQ-9 score of ≥10 was set as the depression threshold [42]. This threshold was established to focus on participants exhibiting at least moderate depressive symptoms, as lower scores typically reflect minimal mood disturbance and are prone to ceiling effects. There were 170 patients with a PHQ-9 score of ≥10 that took the PHQ-9 test at two time points: baseline (Day 0) and post-treatment (Day 31 or greater). Follow-up assessments occurred at least 31 days after baseline. During the study period, a larger number of individuals enrolled in the ReCODE program; however, only participants with baseline PHQ-9 ≥ 10 and a follow-up assessment ≥31 days later were eligible for this analysis. Individuals with PHQ-9 < 10, incomplete baseline data, or shorter follow-up intervals were excluded.

Due to the retrospective nature of the data, exact intervals (mean ± SD) were not uniformly available and could not be statistically modeled. A post hoc sensitivity analysis (G*Power 3.1; two-tailed, α = 0.05, power = 0.95) shows that the available sample (*n* = 170) provides 95% power to detect an effect size of approximately d = 0.28, consistent with small-to-moderate clinical effects. Pre- and post-intervention scores were compared using the non-parametric Wilcoxon signed-rank test, yielding S = 6058, *p* < 0.001, r = 0.46, consistent with a moderate effect size.

## 3. Results

### 3.1. PHQ-9 Score Changes

**Figure 1:**
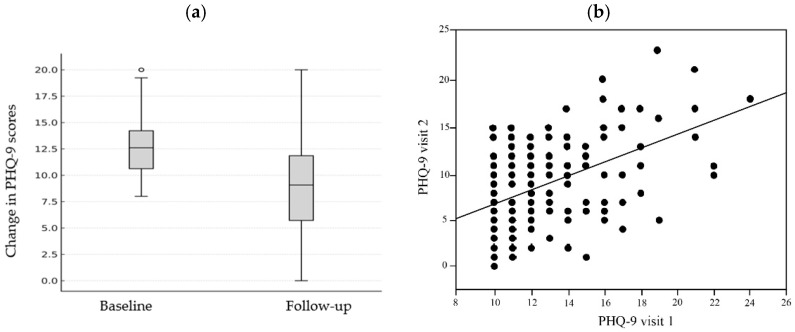
(**a**) Box-and-whisker plot showing PHQ-9 scores at baseline and follow-up in 170 participants who completed ≥31 days of the ReCODE program. Each box represents the interquartile range (IQR) (25th–75th percentile), with the horizontal line indicating the median. The whiskers extend to data points within 1.5 × IQR of the quartiles, while dots represent individual outliers beyond this range. Statistical analysis by Wilcoxon signed-rank test showed a majority (∼80%) of patients exhibiting a reduction in PHQ-9 scores with a mean improvement of 3.96 points (95% CI: 3.34 to 4.57, *p* < 0.0001). (**b**) Bivariate correlation analysis between baseline PHQ-9 scores (visit 1, *x*-axis) and follow-up PHQ-9 scores (visit 2, *y*-axis). The fitted regression line (slope = 0.746, *p* < 0.0001) demonstrates that while baseline depression severity predicts follow-up scores, the slope less than 1.0 indicates proportionally greater improvements among participants with higher initial depression severity.

In a cohort of 170 participants who completed PHQ-9 assessments at two time points spaced at least 31 days apart while adhering to the ReCODE program, there was a statistically significant improvement in depressive symptoms, with approximately 80% (136/170) demonstrating a reduction in PHQ-9 scores (Figure 1a). The mean baseline PHQ-9 score representing moderate depressive symptom severity was 12.64 ± 5.86, indicating moderate depression severity across the study population, decreased to 8.70 ± 5.04 at follow-up, yielding a mean difference of 3.96 points (*p* < 0.0001). This reduction corresponds to a moderate within-subject effect size and reflects clinically meaningful improvement in mood status. Nearly 80% of participants transitioned from the moderate-depression range (10–14) to the mild-depression range (5–9), indicating clinically meaningful change. The 95% confidence interval for the mean difference was 3.34 to 4.57, confirming a consistent direction and magnitude of improvement across the cohort. These results demonstrate a statistically robust and clinically relevant reduction in depressive symptom severity following short-term participation in the ReCODE program.

### 3.2. Baseline Depression Severity and Treatment Response

Bivariate analysis revealed a significant positive correlation between baseline PHQ-9 scores (visit 1) and follow-up scores (visit 2), as shown in Figure 1b, giving a broader view of group-level trends. A linear regression analysis further supports the observed reduction in depressive symptoms following participation in the ReCODE program. Notably, the regression slope of 0.746 (95% CI) was significantly less than 1.0, indicating that participants with higher baseline depression scores experienced proportionally greater absolute improvements. This finding suggests that the magnitude of improvement was more pronounced among those with moderate-to-severe depressive symptoms at baseline, underscoring the program’s responsiveness to initial symptom burden.

## 4. Discussion

Depression represents a critical yet, in the context of multi-modal interventions, underexplored therapeutic target in the management of cognitive decline and early-stage AD. It is a prevalent and often underrecognized comorbidity in AD affecting quality of life, caregiver relationships, and functional independence [31,32]. Depression can exacerbate cognitive symptoms and is itself influenced by neurodegenerative changes, neuroinflammation, neurotransmitter imbalance, and psychosocial stressors [11,33]. The high burden of depression in AD makes it a critical therapeutic target alongside cognition [34,35]. While previous investigations of personalized, multi therapeutic approaches have primarily focused on cognitive outcomes and biochemical parameters [6,10,13,14,16,17,18,19,21,22,23,25], the present study addresses a significant gap by examining depression as a primary endpoint.

The ReCODE program is uniquely positioned to address depression because it intervenes simultaneously on multiple physiological and lifestyle domains implicated in mood regulation. The program’s individualized protocols include: nutritional optimization, glycemic control, hormonal balance, sleep optimization, stress reduction and targeted supplementation [6,10,13,14,16,17,18,19,21,22,23,25].

The results from the present study add to the growing body of evidence from studies employing multi-therapeutic approaches to reverse or delay AD-associated dementia by demonstrating a statistically and clinically meaningful reduction in PHQ-9 depression scores over a short intervention period. These improvements were consistent across baseline severity categories, indicating that benefits were not confined to a single subgroup. The observed one-month improvement in PHQ-9 scores may reflect early physiological effects of improved metabolic control, sleep, and stress reduction, along with enhanced patient engagement. Rapid mood benefits from lifestyle optimization have been reported in prior multimodal interventions [50,51]. When viewed in the context of published SSRI trials, which typically report modest reductions in PHQ-9 scores (−2 to −3 points on average, often without a clinically meaningful shift in severity category) [52,53,54], the magnitude of mood improvement observed in the present study (nearly 4 points) appears clinically noteworthy; however, such comparisons are necessarily indirect given differences in study design, populations, and follow-up duration. As mentioned above, approximately 80% of participants transitioned from moderate to mild depression, highlighting not only statistical but also clinical significance. The robustness of this effect, supported by a narrow confidence interval and regression analysis showing greater benefits among those with more severe baseline depression, suggests that the ReCODE’s multi-therapeutic approach may exert additive or synergistic effects on mood regulating pathways and may offer complementary benefits alongside conventional pharmacotherapy in improving mood-related symptoms within populations at risk for cognitive decline.

It may be argued that improved mood (lower PHQ-9) could cause a pseudo-cognitive improvement and produce better performance on cognitive screening tests without a true change in underlying brain structure or function. Several lines of evidence argue against this interpretation: (1) Studies from our group and other independent researchers have clearly demonstrated objective, test-based cognitive gains (MoCA and computerized neurocognitive batteries) and sustained cognitive improvement in clinical cohorts treated with ReCODE and similar protocols [6,10,13,14,16,17,18,19,21,22,23,25], (2) Improvements have been observed in imaging biomarkers including regional brain volumetrics on MRI—a biological outcome not readily explained by temporary mood shifts [6,10,12,13,14,15,16,17,18,19,21,22,23,24], (3) Lab-based biomarker improvements (e.g., reductions in inflammatory markers and improved metabolic indices) and electrophysiological changes reported in precision-medicine cohorts [6,10,13,14,16,17,18,19,21,22,23,25]. Taken together, these objective findings make it unlikely that the cognitive improvements previously reported by us and others are wholly attributable to an antidepressant effect. Thus, the observation that PHQ-9 scores decreased substantially in our cohort therefore should be interpreted as an expected, clinically important additional benefit of addressing the upstream drivers of neurodegeneration.

The PHQ-9, a validated and widely used depression screening tool, provides a clinically meaningful outcome measure that directly translates to patient well-being and functional capacity [43,44,45,46,47]. Although prior studies from our group and others have emphasized the importance of tracking biochemical parameters, our decision to focus here on depression scores in the present manuscript without including biochemical parameters reflects both methodological considerations and the current state of evidence in this field. The reasons include the following: (1) Previous publications from our research group and others have already established the consistent pattern of metabolic improvements achievable through personalized, multimodal interventions, including reductions in inflammatory markers (hs-CRP), improvements in insulin sensitivity (HOMA-IR), optimization of vitamin D levels, and normalization of glucose metabolism [6,10,13,14,16,17,18,19,21,22,23,25], (2) The present investigation seeks to expand the evidence base beyond metabolic parameters to examine patient-centered outcomes that directly impact quality of life and functional status, (3) Focusing on a single, well-validated outcome measure allows for more rigorous analysis and clearer interpretation of results, avoiding the multiple comparison issues that arise when examining numerous biochemical parameters simultaneously, (4) The PHQ-9’s established clinical significance, with defined thresholds for mild, moderate, and severe depression, enables direct translation of research findings into clinical practice guidelines [28,42,55] and (5) Repeating the biochemical assessments would represent redundant data collection and may detract from the central focus of this paper—namely, the value of the ReCODE program in mood improvement. We therefore reference our earlier work for biochemical outcomes, allowing the present manuscript to serve as a complementary analysis that deepens our understanding of the psychosocial dimensions of cognitive recovery.

Our results underscore the ReCODE program’s capacity to deliver meaningful improvements in mood, in addition to the cognitive, biomarker, and neuroimaging benefits reported in earlier work. This supports the hypothesis that depression is not merely an epiphenomenon of neurodegeneration but a modifiable contributor to disease progression. Therefore, monitoring depression symptoms using validated tools like the PHQ-9 may offer a low-cost, easily administered, and clinically meaningful inclusion in tracking treatment response and disease progression in early-stage AD. As the prevalence of AD rises globally, approaches that simultaneously improve cognition, mood, and systemic health are likely to become increasingly valuable. Future randomized controlled trials are warranted to confirm these results and assess the durability of benefits over longer durations.

## 5. Limitations of the Study

Our study has several limitations inherent to its retrospective design, including incomplete cognitive characterization, variable follow-up intervals, and lack of consistent data on antidepressant use. Although the relationship between mood and cognition is an important research focus, such analysis was not possible here because detailed cognitive measures (e.g., MoCA, MMSE, or CDR scores) were unavailable for all participants. Demographic and cognitive data were inconsistently captured and therefore excluded from this analysis. Likewise, information on concurrent antidepressant or psychotropic medication use was not consistently available; thus, medication initiation or dose changes during follow-up may have contributed to PHQ-9 improvement independent of program effects. Our study relied exclusively on the Patient Health Questionnaire-9 (PHQ-9) as a measure of depressive symptom severity. While the PHQ-9 is a validated and widely used screening instrument, it is not a diagnostic tool for major depressive disorder, and its validity has not been firmly established in individuals with mild cognitive impairment (MCI) or Alzheimer’s disease [28,56,57]. Evidence suggests that in cognitively impaired populations, the PHQ-9 demonstrates good sensitivity (~89%) but only moderate specificity (~71%), indicating an increased likelihood of false positives in this group [58,59]. Consequently, some degree of misclassification bias cannot be excluded, and the self-reported nature of the PHQ-9 may further introduce response bias. Hence, the correlation between baseline and follow-up PHQ-9 scores reflects intra-individual consistency rather than causation.

Furthermore, because participants self-selected into an intensive, clinician-supported program, expectancy effects, social interaction, and nonspecific therapeutic attention may have contributed to observed improvements and cannot be disentangled from program-specific effects. The study also lacked a control group or randomization, limiting causal inference regarding the observed improvements. The one-month reduction in PHQ-9 scores may represent early physiological effects of improved metabolic control, sleep, and stress reduction, as well as enhanced patient engagement; however, the present findings reflect only short-term change and do not permit conclusions regarding the durability or long-term maintenance of benefit, and future controlled studies with longer follow-up durations are warranted.

Given variability in follow-up intervals and incomplete demographic data, covariate-adjusted models could not be implemented. Future prospective investigations warrant clinician-administered assessments (e.g., Geriatric Depression Scale or Neuropsychiatric Inventory) and use mixed-effects modeling to account for time, age, sex, education, and cognitive status to confirm and extend these findings.

## Data Availability

Data for this manuscript were obtained from patient records and anonymized for publication. These data have not been deposited in a public database.
